# Decision Making and Risk Propensity in Individuals with Tendencies towards Specific Internet-Use Disorders

**DOI:** 10.3390/brainsci12020201

**Published:** 2022-01-31

**Authors:** Silke M. Müller, Elisa Wegmann, María Garcia Arías, Elena Bernabéu Brotóns, Carlos Marchena Giráldez, Matthias Brand

**Affiliations:** 1 General Psychology, Cognition and Center for Behavioral Addiction Research (CeBAR), University of Duisburg-Essen, 47057 Duisburg, Germany; elisa.wegmann@uni-due.de; 2 Faculty of Education and Psychology, Universidad Francisco de Vitoria, 28223 Madrid, Spain; maria.garciaarias@ufv.es (M.G.A.); e.bernabeu.prof@ufv.es (E.B.B.); carlosalberto.marchena@ufv.es (C.M.G.)

**Keywords:** internet addiction, gaming disorder, decision making, cognitive functions, impulsivity

## Abstract

The uncontrolled use of specific Internet applications is increasingly recognized as a mental health issue. Gaming disorder, which is one subtype of specific Internet-use disorders (sIUDs), has been included in the ICD-11 as disorder due to addictive behaviors. Addictive disorders are assumed to be accompanied by cognitive deficits as indicated by weaker performance in executive function and risky decision-making tasks. This study investigates risky decision-making in individuals with tendencies towards sIUDs including gaming, online buying-shopping, and social-networks-use disorders. A total of 293 individuals participated in the study. Based on specific screening instruments, the participants were assigned to a group with tendencies towards sIUD or a control group. Participants completed a risky decision-making task and questionnaires assessing risk-taking propensity, impulsivity, psychopathology, and perceived stress. The group with sIUD tendencies showed higher attentional impulsivity and higher levels of depression and anxiety compared to the control group. The groups did not differ in decision making and risk propensity. Decision making did not have significant effects on sIUD symptoms. Risk for developing sIUDs does not appear to be accompanied by altered general decision-making tendencies. Rather, psychological (pre-)load and attentional deficits appear to be relevant features in uncontrolled use of the Internet.

## 1. Introduction

Making advantageous decisions is an everyday challenge. Decisions can, in the short and long term, have beneficial and detrimental consequences. Taken individually, many consequences are hardly noticeable or very subjective. However, they are associated with an assessment of resources to select actions most likely to lead to rewards in the near or distant future [1]. Accumulated decisions represent a decision tendency whose consequences then become noticeable. Addictive behaviors are assumed to represent decision tendencies in which long-term negative consequences are neglected or disregarded in favor of short-term gratification (e.g., [2]). Decisions to perform specific behaviors (e.g., gaming) may be guided by two interacting neural systems: an impulsive system, which is mainly based on associative learning, and a reflective system, which is mainly associated with executive functions and reasoning [3,4,5]. Prefrontal-cortex-related cognitive/inhibitory control is necessary to resist temptations to reach higher-order goals. Thereby, several subcomponents of executive functions are involved in the ability to control urges (e.g., [6]). It is assumed that these cognitive control functions decrease in the course of addiction development while impulsive processes related to limbic structures increase in relevance [2,7,8]. Cognitive deficits in working memory, attention, inhibition, higher-order executive functions, and decision-making have been shown in individuals with substance-use disorders [9,10]. A growing body of research finds similar deficits in neurodegenerative disorders such as Parkinson’s disease [11,12] and in non-substance related disorders, including gambling disorder [13,14] and gaming disorder [15,16]. Gaming disorder is recognized as an official mental disorder in the 11th edition of the *International Classification of Diseases* (ICD-11; [17]) which can occur predominantly online (i.e., on the Internet) or predominantly offline. It is characterized by (a) impaired control over gaming behaviors, (b) increasing priority given to gaming so that it takes precedence over other activities of daily life, and (c) continuation or escalation of the gaming behavior despite negative consequences. Furthermore, the diagnosis requires (d) marked distress and/or functional impairment in important areas of private or professional life. In a similar way, other problematic online behaviors can be classified under the ICD-11 category “other specified disorders due to addictive behaviors”. In particular, problematic online buying-shopping, problematic use of social networks, and problematic online pornography use are potential disorders belonging to this category [18], whereby the latter may also be considered a subtype of Compulsive Sexual Behavior Disorder. Together with gaming disorder, they can be considered specific Internet-use disorders (sIUDs, [19]).

For sIUDs, theoretical frameworks considering the psychological processes involved in addictive behaviors have recently been proposed [20,21,22], which are mainly based on well-known theories of substance-use disorders. According to the I-PACE model, addictive behaviors develop as a result of interactions between predisposing factors, affective and cognitive responses to specific stimuli, and executive functions, such as inhibitory control, that affect decision-making behavior [21]. Diminished general executive functions (crucial for decision making) are assumed to be a risk factor that moderates the relationship between affective responses to triggers (e.g., stress) and the decision to behave in a specific manner. From neuroimaging research on substance-use disorders, Goldstein and Volkow [23] summarized that addiction results from impaired response inhibition and salience attribution (iRISA model). Besides a behavioral tendency towards choosing more risky options, individuals with substance-use disorders show decreased engagement of the salience network (involving the anterior insula, anterior cingulate cortex and inferior parietal lobule) and parts of the executive network (ventrolateral and dorsolateral prefrontal cortex; VLPFC, DLPFC) in non-drug-related decision-making tasks, especially after negative outcomes and during high-risk decisions [24]. Furthermore, addictions are frequently accompanied by impairments in prefrontal cortex functions associated with choosing immediate over delayed gratification and the discounting of future consequences [23]. Disadvantageous decision-making behavior may especially be observed in case of higher emotional arousal such as craving or stress. Regarding sIUDs, meta-analytical findings demonstrate similar deficits in cognitive domains including decision making and executive functions in individuals with problematic Internet use [25]. The effects were found for aggregated studies on different types of sIUDs (i.e., gaming and another online activities), indicating common neurobiological risk factors across different sIUDs [26].

In neuropsychological research, decision making is often measured using gambling tasks that provide uncertain outcomes in terms of gains/losses of fictitious money. One such famous task is the Iowa Gambling Task (IGT; [27,28]) in which participants repeatedly draw a card from one of four decks which results in a gain or loss in each case. Over the course of the task, participants can figure out by the given feedback (gains/losses) that two of the decks are advantageous in the long run and two decks are disadvantageous. In such decision situations, also referred to as “decisions under ambiguous risk”, feedback processing plays a major role, involving brain regions associated with the reward circuit such as the orbitofrontal cortex (OFC) and ventromedial prefrontal cortex (VMPFC), while executive functions become relevant only in the later phases of the task, when the rules become explicit [29]. Similarly, IGT performance is associated with risk taking propensity only in the later trials [30]. One frequently used task with explicit instead of implicit rules is the Game of Dice Task (GDT; [31]). Here, participants bet multiple times on the result of a die roll whereby they have high-risk and low-risk options. In contrast to the IGT, the potential outcomes and their probabilities are explicitly stated or calculable in the GDT. Under these conditions, executive functions associated with fronto-striatal loops are assumed to play a major role in the cognitive and affective processing of feedback [4,32]. Using these tasks, studies reported deficient (i.e., riskier) decision making across various psychopathological conditions (for reviews see e.g., [4,33]) including individuals with tendencies towards sIUDs such as gaming disorder [34,35,36,37] and social-networks-use disorder [38,39] as compared to healthy control groups. However, there were also contradictory findings reporting no significant differences between individuals with sIUD and control groups [40,41,42].

One aspect that is not adequately captured by common neuropsychological decision-making tasks is the devaluation of future compared to immediate consequences—a component of impulsivity which is a common tendency in addictions [43]. The Cards and Lottery Task (CLT; [44]) was especially designed with the goal of simulating the decision-making process in addictions, namely that recurrent preference for immediate reward increases the risk of pronounced negative consequences in the long run. In contrast, forgoing immediate rewards increases the chance of long-term benefits. Unlike other risky decision-making tasks, each decision in the CLT not only has immediate consequences (i.e., direct gain/loss), but also has an impact on future consequences in the sense of an effect on the probability of long-term outcomes occurring (i.e., winning/losing a high amount in the end). Possible immediate and future consequences as well as their risks associated with each decision option are explicitly displayed and vary from round to round. Depending on the current composition of properties, one or the other decision option is more advantageous. Tendentially, the one option (a) is rewarding in the short run but punishing in the long run (i.e., offers relatively high direct gains but increases the risk of losing a large amount in the end) and the other option (b) is not very rewarding or even punishing in the short run but beneficial in the long run (i.e., demands foregoing direct (high) gains but increases the chance of winning a large amount in the end). The CLT thus requires a trade-off between short- and long-term outcomes associated with risks, which goes beyond delay discounting (i.e., a measure of impulsive choice which is also associated robustly with addiction severity, e.g., [43,45]), where decisions are made between sure and immediate versus delayed rewards. The partial feedback version of the CLT additionally maps the asymmetry of the feedback experience. As in most situations in real life, the short-term consequences can be experienced directly, while there is no direct feedback on the extent to which a certain decision has an impact on future consequences. In this CLT version, performance is negatively associated with impulsivity and positively associated with working memory performance, logical reasoning, and need for cognition in the normal population [44]. CLT performance has further been shown to be negatively associated with psychopathological aspects such as symptoms of depression, difficulties in emotion regulation, severe obesity, and symptoms of attention-deficit/hyperactivity disorder [46]. In a recent study on social-networks-use disorder, we found that individuals with problematic social-networks use showed deficits in executive functions, but not in CLT performance [47]. Whether biases in general decision-making tendencies to prefer short-term outcomes by neglecting negative effects on future consequences and/or differences in general propensity to take risks apply to sIUDs remains to be explored.

Common attributes associated with sIUDs are certain predisposing individual characteristics, such as (comorbid) psychopathology and temperamental features [21]. Symptoms of depression and anxiety were shown to be associated with gaming disorder [48], social networks-use disorder [49], and buying-shopping disorder [50]. High impulsivity (as a temperamental feature) has been associated with unspecified Internet-use disorder [51] but also with gaming disorder [16,52], social-networks-use disorder [53,54], and buying-shopping disorder [55]. According to ICD-11, the latter might most likely be classified as a specified impulse control disorder, but there are reasonable arguments to classify it as a behavioral addiction [18,50,56]. For example, neuroimaging studies suggest the involvement of reward pathways in buying-shopping disorder, which suggests addiction-like processes [57]. It is apparent that there is some overlap between impulse control disorders and addictive disorders including sIUDs, e.g., both involve inhibitory control [58]. However, the mere involvement of similar components does not necessarily imply similar psychological mechanisms across disorders [21]. In the I-PACE model, Brand and colleagues [21] proposed that deficient general inhibitory control serves as a moderator of the relationship between affective/cognitive responses to triggering stimuli (e.g., stress) and the decision to perform a specific behavior. In general, the model assumes addictive disorders result from interactions between personal characteristics and certain moderating and mediating variables. In line with this, for example, we found that interactions between impulsivity and general executive functions explained the symptom severity of a social-networks-use disorder [53]. It remains to be determined whether general risky decision tendencies have a similar moderating effect.

This study aimed to investigate decision making and risk propensity in sIUDs. As previous findings indicate common neurobiological risk factors across different types of sIUDs [25,26] we considered individuals with symptoms aggregated across different types of (potential) sIUDs, namely gaming disorder, online buying-shopping disorder, and social-networks-use disorder. We had the following hypotheses:

1. Individuals with tendency towards sIUD differ from those with regular/non-problematic use of the Internet regarding risky decision-making.

Based on the I-PACE model, we further assume that decision making and certain personal characteristics interactively contribute to sIUD development. These interactions might be already present in early stages but may especially occur in later stages of the disorder (i.e., when symptom severity is high). Accordingly, we considered potential interaction effects exclusively within the group including participants with sIUD symptoms (because the control group, by definition, had no variance in symptom severity). Our second hypothesis was:

2. Risky decision-making moderates the effect of (a) trait impulsivity, (b) psychopathological symptoms, and (c) perceived stress on sIUD symptom severity.

Additionally, based on previous findings, we assume individuals with tendency towards a sIUD to show higher (a) trait impulsivity, (b) psychopathological symptoms, and (c) perceived stress compared to those with regular/non-problematic Internet use. Risky decision-making was operationalized by a behavioral measure (i.e., CLT behavior) and a self-reported measure of risk propensity to differentiate behavior in the rather complex task from general risk seeking tendency.

## 2. Materials and Methods

Due to restrictions associated with the COVID-19 pandemic, the study was conducted online in Germany and Spain in the period from 16 February 16 to 14 May 2021. A total of 347 individuals completed the online study. Outliers in terms of age (*n* = 21) and individuals who had indicated that they had technical problems with the integrated decision-making task (*n* = 33) were excluded. The final sample consisted of 293 individuals (228 female, 63 male, 2 non-binary) between 18 and 34 years of age, *M* = 20.85, *SD* = 2.92. All participants were active Internet users. Most of the participants (78.8%) had general university entrance qualification as highest level of education. The majority of participants (93.2%) indicated use of social networks, about two thirds (67.2%) indicated shopping online, and about one fifth (21.8%) of the participants indicated playing online games regularly. Based on the scores of the screening instruments used, individuals were assigned to the “sIUD tendency” group if they were showing at least one screened symptom of a sIUD and to the control group if they were showing no sIUD symptoms. Individuals who could not be clearly assigned to one group because either they did not use any of the specified activities (*n* = 8) or in which no predominant sIUD could be identified (*n* = 4) were excluded. All participants provided informed consent prior to participation. The study was approved by the local ethics committee.

We used the 10-item Internet Gaming Disorder Test (IGDT-10; [59]) in translated and different adapted versions as screener for sIUD symptom severity. The IGDT-10 operationalizes the nine criteria for Internet gaming disorder mentioned in the fifth edition of the *Diagnostic and Statistical Manual of Mental Disorders* (DSM-5; [60]). Besides the original version for gaming, we used two adapted versions for measuring social-networks-use disorder and online buying-shopping disorder. In the adapted versions, we exchanged the term “gaming” for the other online behavior (i.e., “social networking” and “online shopping”) respectively. Each item was rated on a 3-point scale (0 = “never”, 1 = “sometimes”, 2 = “often”). As described by Király and colleagues [59], a criterion is met (i.e., scores 1) if the response was ”often”. Each item represents one criterion, except for items 9 and 10, only one of which must be answered ”often” to fulfill the criterion. The sum score can range between 0 and 9, with a score ≥ 5 indicating clinical relevance [59]. Participants conducted the specific IGDT-10 for all behaviors previously indicated to be done on a regular basis. The IGDT-10 scores were used to assign individuals to the “at-risk” group and the control group, respectively. Any IGDT-10 score above 0 (i.e., meeting at least one criterion) indicates that the individual is at risk of developing an sIUD. The control group was determined by IGDT-10 scores of 0, which includes individuals who indicated at least one of the investigated online behaviors (i.e., gaming, shopping, social networks use) but did not meet any of the DSM-5 criteria. For the “at-risk” group, the IGDT-10 scores were aggregated into one score (“IGDT-10agg”) indicating the symptoms/fulfilled criteria of the predominant sIUD. In case individuals were at risk for more than one sIUD, the predominant sIUD was determined by either the highest IGDT-10 score or (if scores were equal) by the number of fulfilled criteria described as being particularly relevant [61]. The IGDT-10 versions had adequate reliability in the current sample with Cronbach’s α scores of 0.827 (gaming), 0.794 (shopping) and 0.770 (social networking).

**Figure 1:**
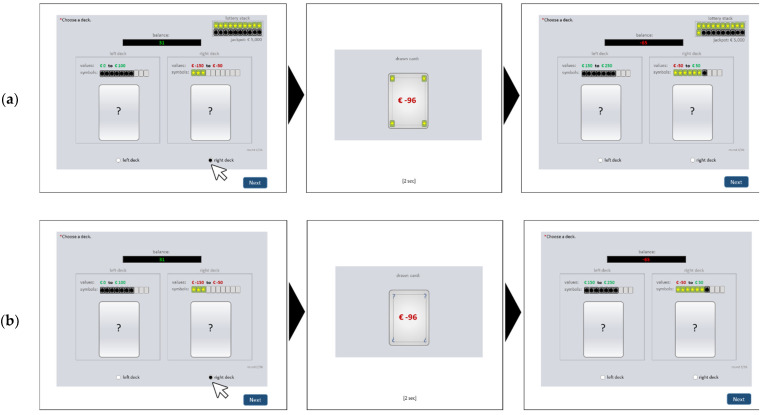
Exemplary trial in the online version of the Cards and Lottery Task (CLT). In this example, in round 2 (left screen), the right deck is chosen. It contains ten cards with negative values between −150 to −50, and three of the cards have a star symbol. The drawn card (middle screen) has a value of −96 and a star symbol. The value was offset against the current account balance, leading to a new balance of −65 in round 3 (right screen). The star was added to the long-term account, “lottery stack”, which increased the chance to win the jackpot at the end. In the next round, both decks contain ten new cards each. Accordingly, the value ranges and/or contained number of cards with star/bomb symbol change. In the current example (right screen) the left deck now contains cards with values between 150 to 250 and seven bomb-cards; the right deck now contains cards with values between −50 and +50 with six cards of the cards having a star and one card having a bomb symbol. The example is shown for two feedback variations: (**a**) full feedback is provided by showing the drawn card’s value and symbol, as well as the actual stats of the lottery stack (as in the five trial rounds); (**b**) partial feedback is provided by showing only the short-term effects and hiding the drawn card’s symbol and the lottery stack (as in the actual task). For a more detailed description of the CLT see [44].

An online version of the Cards and Lottery Task (CLT; [44]) was used as a behavioral measure of risky decision-making. The CLT is a computerized gambling task which reflects basic assumptions of decision making in addictions, namely that that frequent choice of high immediate rewards increases the risk for serious negative consequences in the future. To map this interplay, in the CLT (in contrast to other risky decision-making tasks) each decision option had short-term consequences and long-term consequences, which were in conflict with each other. In each of 36 rounds, the participant has to choose between two decision options (i.e., two decks including ten cards each that were renewed each round). Each choice resulted in an outcome (i.e., one card drawn randomly from the chosen deck) that had two properties: a short-term consequence (i.e., a positive/negative value leading to an immediate gain/loss of virtual money) and a long-term consequence (i.e., a star or bomb symbol leading to an increase/decrease of the probability of winning a high amount of virtual money in a lottery at the end of the game). The decision outcomes were cumulated into a short-term account (where the card value was directly added/subtracted) and a long-term account (with the ratio of stars/bombs representing the chance to win/lose a high amount in the lottery). The state of the long-term account (i.e., ratio of stars and bombs) determined the probability of positive/negative long-term effects (i.e., winning/losing the lottery; win: jackpot is added; loss: jackpot is subtracted). Throughout the game, explicit information about possible short- and long-term consequences (i.e., possible value range and amounts of cards with stars/bombs included in each deck) was displayed for each of the two options. The contingencies changed from round to round but the two decision options vary systematically in terms of the possible consequences: one option (“short-term deck”, left) tended to lead to high immediate gains (i.e., contains cards with high positive values), but increased the risk of negative long-term consequences (i.e., contained many cards with bomb symbol). The other option (“long-term deck”, right) tended to lead to only small immediate gains or even losses (i.e., contained cards with low positive or negative values) but increased the chance of positive long-term consequences (i.e., many cards with the star symbol). Figure 1 shows an exemplary round. Due to the explicitness of possible outcomes, the CLT reflects decisions under objective risk. For the purpose of this online study, we implemented the CLT within an online survey tool. Every participant conducted the CLT in the partial feedback version. In this version, after each choice, feedback about the outcome (i.e., the drawn card) was presented, but only the short-term consequence (i.e., value) was shown, while the long-term consequence (i.e., symbol) was hidden (see Figure 1b). This version most closely represents situations in everyday life, where in most cases only the short-term effects of a decision are salient. It was used previously in a laboratory setting in the context of problematic social networks-use [47]. In this study, we used the CLT net score (i.e., n of choices for the long-term deck minus n of choices for the short-term deck) with possible values between -36 and 36. More negative values represent a stronger tendency to prefer immediate rewards by neglecting long-term risks.

The Risk-Propensity Scale (RPS; [62]) was used as a self-report measure of risky decision-making tendency. It consists of seven items answered on a nine-point Likert scale ranging from 1 (“totally disagree”/“risk avoider”) to 9 (“totally agree”/“risk seeker”). Higher mean scores indicate a greater tendency to take risks. The internal consistency in our sample was α = 0.785.

The 15-item Barratt Impulsiveness Scale (BIS-15) in the German [63] and Spanish [64] versions was used to measure trait impulsivity. Each item was rated on a four-point Likert scale from 1 (“rarely/never”) to 4 (“almost always/always”). The scale comprises the three dimensions of non-planning, motor, and attentional impulsivity, with higher scores indicating greater impulsiveness. The overall internal consistency in our sample was α = 0.823 for the German version and α = 0.820 for the Spanish version.

The Brief Symptom Inventory (BSI; [65]) is a measure of subjective impairment due to psychological symptoms, consisting of 53 items. For this study, we used the subscales for depression and anxiety, each comprising 6 items. Participants indicate their level of agreement by means of a five-point Likert scale ranging from 0 (“not at all”) to 4 (“extremely”). Internal consistency in our sample was good, with Cronbach’s α = 0.849 for the depression subscale and α = 0.842 for the anxiety subscale.

The Perceived Stress Scale (PSS) originally developed by Cohen, et al. [66] was used in the German [67] and Spanish [68] ten-item versions. Participants indicated the frequency of certain feelings and thoughts in the last month on a five-point Likert scale from 1 (“never”) to 5 (“very often”). The sum score served as a global measure of perceived stress during the last month with higher scores reflecting greater stress levels. Because the survey took place during a period when acute restrictive measures were in effect to contain the COVID-19 pandemic, we additionally assessed perceived strain due to COVID-19-related restrictions with an extended version of the questionnaire by Wegmann, et al. [69]. The items were adapted/expanded to meet the restrictions prevailing at the time. Participants were asked to indicate their subjectively perceived strain for each of 22 different restrictions (e.g., “Contact prohibitions”, “Closing of stores”, “Unemployment/financial losses”, or “Illness of relatives/friends/acquaintances”) on a five-point Likert scale from 1 (“not strained at all”) to 5 (“very strongly strained”). We calculated the mean over all items. A higher score indicates higher perceived strain. With Cronbach’s α = 0.899, the scale showed high internal consistency.

Statistical analyses were carried out using SPSS Statistics 26 [70]. In case of variance heterogeneity, the Welch correction was used. Moderated regression analyses were used to test interaction effects. Predictor variables were mean-centered before analysis.

## 3. Results

### 3.1. Descriptive Statistics

The ”sIUD tendency” group (*n* = 174; 143 female, 31 male, 0 non-binary) and the control group (*n* = 107; 76 female, 29 male, 2 non-binary) differed with regard to age (*t*(164) = 1.99, *p* = 0.049) and gender (χ^2^ (1, 281) = 16.00, *p* < 0.001). The “sIUD tendency” group was slightly younger (age: *M* = 20.57, *SD* = 2.39) and included a higher proportion of females (82%) as compared to the control group (age: *M* = 21.36, *SD* = 3.61; 71% females). Table 1 shows the descriptive statistics of the main variables per group.

Within the “sIUD tendency” group, the IGDT-10agg scores ranged between 1 and 9, with a mean IGDT-10agg score of 2.42 (*SD* = 1.57). From the 174 individuals in the “sIUD tendency” group, 13 (7.5%) were above the described cut-off for clinical relevance.

### 3.2. Group Comparisons

To test our first hypothesis, we calculated differences between groups regarding measures of risky decision making. In addition, we calculated differences with respect to the other variables (see Table 1). We accounted for age and gender differences between the groups by using MANCOVAs, including age, as a covariate and gender as additional factor.

#### 3.2.1. Differences in Risky Decision-Making

The “sIUD tendency” group showed a slightly lower mean CLT net score than the control group. However, the effect was not significant (see Table 1). Likewise, the groups did not differ significantly in risk propensity (see Table 1). Accordingly, individuals at risk of an sIUD showed neither altered decision-making tendencies nor stronger tendencies to take risks. The factor of gender had a significant effect on risk propensity, *F*(2,275) = 5.26, *p* = 0.006, η^2^_p_ = 0.037, with higher scores for males (*M* = 4.48, *SD* = 1.35) than for females (*M* = 3.81, *SD* = 1.32) and non-binary individuals (*M* = 3.64, *SD* = 0.30). There were no significant interactions between group and gender.

#### 3.2.2. Differences in Impulsivity

The “sIUD tendency” group and the control group did not differ in terms of non-planning and motor impulsivity, but individuals with tendencies towards an sIUD showed higher attentional impulsivity as compared to the control group (see Table 1). Gender effects were significant only for non-planning impulsivity, *F*(2,275) = 3.58, *p* = 0.029, η^2^_p_ = 0.025. There were no interaction effects.

#### 3.2.3. Differences in Psychopathology

The “sIUD tendency” group showed significantly higher severity for symptoms of depression and anxiety than the control group, as indicated by the respective BSI sub scores (see Table 1). The covariate of age had a significant effect on depression (*F*(1,275) = 5.93, *p* = 0.016, η^2^_p_ = 0.021). There were no significant interaction effects.

#### 3.2.4. Differences in Perceived Stress

Individuals with tendencies towards an sIUD perceived significantly more stress and greater COVID-19-related burden than individuals with regular/non-problematic Internet use (see Table 1). Main effects of gender were present for both perceived stress (*F*(2,275) = 7.46, *p* = 0.001, η^2^_p_ = 0.051) and COVID-19-related strain (*F*(2,275) = 15.71, *p* < 0.001, η^2^_p_ = 0.103). Females reported higher stress (*M* = 31.42, *SD* = 6.57) and burden from COVID-19 containment measures (*M* = 3.14, *SD* = 7.2) as compared to males (PSS: *M* = 28.18, *SD* = 8.18; COVID-19 strain: *M* = 2.42, *SD* = 0.68). The covariate of age also had significant effects on perceived stress (*F*(1,275) = 11.91, *p* = 0.001, η^2^_p_ = 0.086) and COVID-19-related strain (*F*(1,275) = 21.07, *p* < 0.001, η^2^_p_ = 0.071) variables. There were no interaction effects.

### 3.3. Exlpanation of Variance in Symptom Severity within the “sIUD Tendency” Group

To test our second hypothesis, we considered linear associations within the group of individuals with sIUD tendencies. In preparation for the moderated regression analyses, we first analyzed correlations of the main variables with the measure of symptom severity (i.e., IGDT-10agg).

#### 3.3.1. Bivariate Correlations

The results (see Table 2) show that symptom severity of an sIUD did not correlate with general tendencies to prefer long-term over short-term rewards in the CLT. But the small positive correlation with risk propensity indicated that higher propensity to take risks partly goes along with more severe sIUD symptoms. Furthermore, sIUD symptom severity was associated with increased motor and attentional impulsivity, as well as with higher levels of depression, anxiety, perceived stress, and COVID-19-related strain (see Table 2).

The association between CLT net score and risk propensity was in the expected negative direction (indicating that higher risk-taking tendency leads to less advantageous decision making in the CLT), but not statistically significant. The CLT net score showed an expected negative correlation with impulsivity (i.e., non-planning), indicating that higher impulsiveness goes along with a tendency to prefer short-term gains by neglecting negative long-term outcomes. Moreover, as expected, (a) risk propensity correlated positively with measures of impulsivity, (b) psychopathological symptoms were intercorrelated and showed high positive correlations with perceived stress (see Table 2).

#### 3.3.2. Moderated Regression

We calculated moderated regression analyses within the “sIUD tendency” group to analyze the assumed interaction effects. In all moderation analyses, symptom severity (IGDT-10agg) was the dependent variable. The predictors were: impulsivity facets (BIS-15: non-planning, motor, attentional), psychopathological symptoms (BSI: depression, anxiety), and perceived stress (PSS and COVID-19 strain). Risky decision-making tendencies as indicated by decision-making behavior in the CLT (CLT net score) and risk propensity (RPS mean) served as moderators. Combinations of the different predictors and moderators were tested in individual analyses.

Significant overall effects were obtained for the models including BIS-15 motor, BIS-15 attentional, BSI depression, BSI anxiety, PSS, and COVID-19 strain (see Table 3). In all models, the respective predictor had a significant effect on the dependent variable. CLT performance and risk propensity had no moderating effects. However, risk propensity had incremental effects explaining additional 2 to 3% of the variance in symptom severity beyond the effects of attentional impulsivity (model 4, Δ*R*^2^ = 0.020), anxiety (model 8, Δ*R*^2^ = 0.036), perceived stress (model 9, Δ*R*^2^ = 0.029), and COVID-19-related strain (model 10, Δ*R*^2^ = 0.033), respectively. CLT performance did not have any significant effect on sIUD symptom severity.

## 4. Discussion

This study investigated decision-making and risk propensity in sIUDs. Risky decision-making tendencies were assessed with a behavioral measure (CLT) and a self-report measure (RPS) that were compared between individuals with a tendency towards an sIUD and individuals with regular/non-problematic Internet use. Other common predictors, including impulsivity, psychopathology, and stress vulnerability were additionally analyzed. Within individuals with sIUD tendency, we further examined correlations with aggregated sIUD symptom severity and analyzed potential interactions between the mentioned predictors and risky decision-making tendencies.

The “sIUD tendency” group and the control group did not show any significant differences in risky decision-making tendencies. This was against our hypothesis. However, the results were in line with those of a previous study in which we did not find significant differences in CLT performance between individuals with and without tendencies towards social-networks-use disorder [47]. Studies using other risky decision-making tasks reported riskier decision making in individuals with tendencies towards sIUD or gaming disorder specifically [34,35,36,37,38,39]. However, other studies reported no behavioral differences [40,41,42]. Decision making and related cognitive processes, such as working memory and attention, are considered impaired in addictive disorders [23,25]. However, individuals with substance-use disorder also show reduced sensitivity towards non-addiction-related reinforcers, as indicated, for example, by reduced VLPFC activation during monetary gain or loss [23]. Reduced sensitivity to monetary losses has also been demonstrated in individuals with sIUDs (e.g., [71]). Unlike in other tasks, such as the IGT, in the CLT, high loss sensitivity went along with worse performance [46]. Correspondingly, reduced loss sensitivity can have beneficial effects on CLT performance (as choosing the beneficial option sometimes comeswith immediate losses), which could potentially compensate for deficits arising from weaknesses in cognitive functions in individuals with sIUDs.

In individuals with tendencies towards an sIUD, symptom severity did not correlate significantly with CLT performance. Depression, anxiety, and attentional impulsivity were most strongly associated with sIUD symptoms, which supports previous findings (e.g., [49,72]). However, symptom severity was also positively associated with self-reported risk propensity. The results indicate that the general tendency to take risks contributes (to a small but significant extent) to the explanation of sIUD symptoms. More precisely, risk propensity may add an increment to the effects of attentional impulsivity, anxiety, and perceived (current) stress, as indicated by the results of the regression analyses. In accordance with the I-PACE model [20,21], general risk propensity can be categorized as a core characteristic that adds to other predisposing variables including psychopathology and other temperamental features.

Against our hypotheses, risky decision-making tendencies did not interact with impulsivity, psychopathological symptoms, or stress vulnerability in the prediction of sIUD symptoms. According to process models of addiction, disadvantageous decision-making behavior may especially be observed in situations with higher emotional charge, such as when triggers are present that induce certain cognitive and affective responses, i.e., craving and urges [21,23]. Regarding sIUDs, studies on online buying-shopping disorder and social-networks-use disorder that used decision-making tasks with addiction-related stimuli indicate that cue-reactivity and craving interfere with decision quality [73,74]. In this study, we did not induce craving, nor did we use addiction-related stimuli. This may be the reason why we could not find the suspected behavioral tendency. This fits with the argumentation (e.g., within the I-PACE model) that it might be adequate to distinguish between general executive functions and stimuli/situation-specific reductions of executive functions. Future studies may add addiction-related stimuli to the CLT or may induce craving before performing the CLT to better understand the potential impact of situation-specific reductions of decision making in individuals with sIUDs. Furthermore, the effects might be clearer in patients with pathological use of specific internet applications, which might be overlooked in a group including individuals with only few symptoms.

A clear limitation of our study was that it was conducted in a non-controlled online setting. This was applied due to prevailing contact restriction policies during the COVID-19 pandemic. Despite thorough exclusion of non-valid-seeming data sets, behavior in the decision task may have been affected by this data collection method in particular. However, the mean CLT net score in both groups was considerably higher than reported for the laboratory condition [44,75], which indicates that there were no difficulties in understanding. Future studies should validate the online CLT version in a direct comparison with the original version conducted in a laboratory. Measures of related constructs such as inhibitory control or other executive function components could not be implemented in this study design. Accordingly, in this study we focused on mapping decision tendencies in more complex decision situations rather than capturing multiple process components, which is contrary to other frameworks (e.g., [76]). Future studies should further investigate the involvement of specific executive functions in sIUDs, including inhibition and its component processes as well as gain-/loss sensitivity and its corresponding neural correlates. Inhibitory control, defined as the ability to suppress processing of prepotent maladaptive responses, plays a crucial role in impulse control disorders [6]. In case an sIUD cooccurs with impulsive/compulsive disorders, inhibitory control and decision making appear to be impaired, but not if the sIUD occurs without comorbid impulsive/compulsive disorders [58]. Similarly, patients with Parkinson’s disease and comorbid impulse control disorders showed decreased performance in set-shifting and reward-related decision-making tasks [77]. Determining under which conditions maladaptive decision-making behavior occurs and whether it results more from an impaired ability to control impulses and/or from altered sensitivity to (non-addiction-related) reinforcers is crucial to characterize the phenomena. Identifying the mechanisms behind specific disorders is essential to develop effective prevention and intervention measures. Nevertheless, the CLT offers the chance to map complex decision tendencies that are assumed to underlie addictive behavior in a way that no other task does so far.

Taken together, the current findings indicate that tendencies for sIUDs are not significantly associated with a general tendency to prefer short-term gratification by neglecting long-term risks. Rather, mental condition and impulsivity appear to be risk factors, but so does general risk-taking propensity, albeit to a smaller extent. The latter is more likely to be covered by other gambling tasks such as the GDT, while aspects of impulsivity are more likely covered by delay discounting tasks [78]. Choosing immediate gratification, besides knowing that this may have negative effects in the future as operationalized by the CLT, does not seem to be a general decision-making style for people with addictive tendencies, but seems to apply only/especially to the specific addiction-associated behavior. 

## Figures and Tables

**Table 1:** Descriptive statistics and group comparisons.

	sIUD Tendency (*n* = 174)		Control (*n* = 107)		Comparison		
Variable	*M*	(*SD*)	*M*	(*SD*)	*F*(1,275)	*p*	Partial Eta ^2^
CLT net score	6.11	(13.42)	7.96	(14.85)	0.37	0.545	0.001
RPS (mean) ^b^	3.90	(1.34)	4.04	(1.37)	0.71	0.399	0.003
BIS-15 non-planning (mean) ^b^	2.24	(0.62)	2.25	(0.64)	0.03	0.867	<0.001
BIS-15 motor (mean)	2.07	(0.59)	1.93	(0.50)	0.79	0.376	0.003
BIS-15 attentional (mean)	2.39	(0.66)	2.13	(0.57)	10.20	0.002	0.036
BSI: Depression (mean) ^a^	1.36	(0.90)	0.92	(0.79)	19.65	<0.001	0.067
BSI: Anxiety (mean)	1.29	(0.91)	0.86	(0.66)	14.45	<0.001	0.050
PSS (sum) ^a,b^	32.32	(6.61)	28.16	(7.01)	25.75	<0.001	0.086
COVID-19 strain (mean) ^a,b^	3.11	(0.72)	2.77	(0.80)	15.71	<0.001	0.103

^a^ significant effect of age; ^b^ significant effect of gender; CLT = Cards and Lottery Task (net score possible range: −36 to 36); RPS = Risk Propensity Scale (possible range: 1 to 9); BIS-15 = Barratt Impulsiveness Scale 15 item version (possible range: 1 to 4); BSI = Brief Symptom Inventory (possible range: 0 to 4); PSS = Perceived Stress Scale (possible range: 10 to 50); COVID-19 strain = perceived strain due to the emergence of the coronavirus disease 2019 (possible range: 1 to 5).

**Table 2:** Coefficients of the correlations with symptoms of a specific Internet-use disorder (IGDT-10agg) within the “sIUD tendency” group.

		1	2	3	4	5	6	7	8	9
1	IGDT-10agg	-								
2	CLT net score	−0.028	-							
3	RPS	0.180 *	−0.110	-						
4	BIS-15 non-planning	0.125	−0.236 **	0.370 **	-					
5	BIS-15 motor	0.257 **	−0.037	0.274 **	0.337 **	-				
6	BIS-15 attentional	0.300 **	−0.053	0.128	0.361 **	0.366 **	-			
7	BSI: Depression	0.305 **	−0.024	0.188 *	0.166 *	0.238 **	0.352 **	-		
8	BSI: Anxiety	0.287 **	0.041	−0.037	0.045	0.143	0.366 **	0.646 **	-	
9	PSS	0.172 *	0.108	0.055	0.035	0.280 **	0.396 **	0.673 **	0.517 **	-
10	COVID-19 strain	0.170 *	−0.098	−0.012	−0.019	0.058	0.097	0.014	0.249 **	0.034

N = 174; * *p* < 0.05; ** *p* < 0.01 (two-tailed); IGDT-10agg = ten item Internet Gaming Disorder Test aggregated score indicating symptom severity of the predominant risky Internet-use behavior; CLT = Cards and Lottery Task; RPS = Risk Propensity Scale; BIS-15 = 15-item Barratt Impulsiveness Scale; BSI = Brief Symptom Inventory; PSS = Perceived Stress Scale; COVID-19 = coronavirus disease 2019.

**Table 3:** Statistics of significant moderated regression models predicting sIUD symptoms.

		Coefficients			Overall Model Results		
Model	Predictors	β	*T*	*p*	*F*	*p*	*R* ^2^
1	BIS-15 motor (mean)	0.257	30.48	0.001	4.44	0.005	0.073
	CLT net score	−0.027	−0.365	0.716			
	Interaction	0.080	10.08	0.281			
2	BIS-15 motor (mean)	0.202	20.60	0.010	5.69	0.001	0.091
	RPS (mean)	0.114	10.50	0.136			
	Interaction	0.113	10.52	0.132			
3	BIS-15 attentional (mean)	0.281	30.82	<0.001	6.54	<0.001	0.103
	CLT net score	−0.009	−00.12	0.904			
	Interaction	0.117	10.59	0.114			
4	BIS-15 attentional (mean)	0.277	30.67	<0.001	7.05	<0.001	0.111
	RPS (mean)	0.146	10.99	0.048			
	Interaction	0.020	00.26	0.795			
5	BSI: Depression (mean)	0.305	40.18	<0.001	5.99	0.001	0.096
	CLT net score	−0.020	−00.28	0.784			
	Interaction	0.050	00.68	0.497			
6	BSI: Depression (mean)	0.276	30.72	<0.001	6.97	<0.001	0.110
	RPS (mean)	0.125	10.69	0.093			
	Interaction	0.035	00.48	0.633			
7	BSI: Anxiety (mean)	0.291	30.97	<0.001	5.40	0.001	0.087
	CLT net score	−0.035	−00.48	0.632			
	Interaction	0.054	00.73	0.467			
8	BSI: Anxiety (mean)	0.289	40.01	<0.001	7.90	<0.001	0.122
	RPS (mean)	0.196	20.72	0.007			
	Interaction	0.060	00.83	0.411			
9	PSS (sum)	0.158	20.11	0.037	3.72	0.013	0.062
	RPS (mean)	0.170	20.29	0.023			
	Interaction	0.053	00.71	0.477			
10	COVID-19 strain (mean)	0.173	20.32	0.021	3.75	0.012	0.062
	RPS (mean)	0.183	20.45	0.015			
	Interaction	0.006	00.08	0.935			

N = 174; sIUD = specific Internet-use disorder; CLT = Cards and Lottery Task; RPS = Risk Propensity Scale; BIS-15 = Barratt Impulsiveness Scale 15 item version; BSI = Brief Symptom Inventory; PSS = Perceived Stress Scale.

## Data Availability

The data are available from https://osf.io/ayu7z/ (accessed on 31 January 2022).
